# Cumulative asparagine to aspartate deamidation fails to perturb γD‐crystallin structure and stability

**DOI:** 10.1002/pro.5120

**Published:** 2024-07-18

**Authors:** Alex J. Guseman, Jeremy J. González, Darian Yang, Angela M. Gronenborn

**Affiliations:** ^1^ Department of Structural Biology University of Pittsburgh School of Medicine Pittsburgh Pennsylvania USA

**Keywords:** deamidation, dynamic light scattering, human γD‐crystallin, molecular dynamics, NMR

## Abstract

Deamidation frequently is invoked as an important driver of crystallin aggregation and cataract formation. Here, we characterized the structural and biophysical consequences of cumulative Asn to Asp changes in γD‐crystallin. Using NMR spectroscopy, we demonstrate that N‐ or C‐terminal domain‐confined or fully Asn to Asp changed γD‐crystallin exhibits essentially the same ^1^H‐^15^N HSQC spectrum as the wild‐type protein, implying that the overall structure is retained. Only a very small thermodynamic destabilization for the overall Asn to Asp γD‐crystallin variants was noted by chaotropic unfolding, and assessment of the colloidal stability, by measuring diffusion interaction parameters, yielded no substantive differences in association propensities. Furthermore, using molecular dynamics simulations, no significant changes in dynamics for proteins with Asn to Asp or iso‐Asp changes were detected. Our combined results demonstrate that substitution of all Asn by Asp residues, reflecting an extreme case of deamidation, did not affect the structure and biophysical properties of γD‐crystallin. This suggests that these changes alone cannot be the major determinant in driving cataract formation.

## 1 INTRODUCTION

Cataracts form when the crystallin proteins of the eye lens aggregate, causing opacification of the lens and subsequent visual impairment (Lam et al., 2015). The eye lens is a unique organ that specifically evolved to reduce light scattering and focus the incoming image onto the retina (Bloemendal et al., 2004). As the lens develops in utero, all members of the crystallin superfamily of proteins are synthesized in the lens fiber cells in defined proportions, reaching a final overall concentration >400 g/L. Subsequently, all other macromolecules and organelles that scatter light are removed. As a result, mature lens fiber cells are essentially a membrane bound sack of crystallin proteins that lack the ability to synthesize or degrade proteins (Bloemendal et al., 2004; Lynnerup et al., 2008; Sharma and Santhoshkumar, 2009). Therefore, crystallin proteins must remain stable and soluble over the lifetime of an organism or, if not, increase the likelihood of cataractogenesis and visual impairment.

Crystallin proteins comprise 95% of the protein mass in mature lens fiber cells and are divided into three subgroups, α, β, and γ crystallins. Structurally homologous, the β/γ‐crystallins are composed of two β‐sheet domains with Greek key motifs (Basak et al., 2003; Ghosh and Chauhan, 2019; Jaenicke and Slingsby, 2001). They are thermodynamically very stable and highly soluble. The individual domains are connected by a flexible linker, with the β‐crystallins possessing an extended linker and oligomerization propensity (Bloemendal et al., 2004; Lampi et al., 2014; Xi et al., 2017), whereas the γ‐crystallins possess a shorter linker and are monomeric. The α‐crystallins are small heat‐shock proteins that form oligomeric complexes and function as chaperones for the β/γ crystallins (Haslbeck et al., 2016; Jehle et al., 2010; Jehle et al., 2011). Even while endowed by evolution with unique biophysical properties and chaperoned by dedicated α‐crystallins, decades of exposure to environmental stresses and spontaneous chemical reactions can result in covalent modifications, such as deamidation, truncation, phosphorylation, oxidation as well as others, that may have detrimental effects on the structure and stability of β/γ crystallins (Hains and Truscott, 2010; Sharma and Santhoshkumar, 2009).

Deamidation is a spontaneous chemical reaction converting asparagine (Asn)/glutamine (Gln) side chains into aspartic acid (Asp)/glutamate (Glu) or iso‐aspartic/iso‐glutamic acid (Clarke, 1987; Geiger and Clarke, 1987). Proteomic analyses of aged lenses have identified deamidation of Asn and Gln as a common modification of aged crystallin proteins, thus implicating deamidation as a possible driver of cataract formation (Hains and Truscott, 2010; Hanson et al., 2000; Lampi et al., 1998). While proteomics identified equal amounts of Asn and Gln deamidation in lens tissue (Hains and Truscott, 2010), Gln deamidation occurs at a much slower rate in model systems (Robinson and Robinson, 2001), leading to an increased focus on Asn deamidation. Numerous studies have been conducted to investigate how Asn deamidation influences the structure and biophysical properties of β/γ crystallins (Flaugh et al., 2006; Forsythe et al., 2019; Guseman et al., 2021; Kim et al., 2002; Lampi et al., 2006; Lampi et al., 2014; Michiel et al., 2010; Pande et al., 2015; Ray et al., 2016; Takata et al., 2008; Takemoto and Boyle, 2000; Vetter et al., 2020). These studies were carried out using recombinantly expressed crystallin proteins that possess Asn to Asp changes as surrogates for site specific deamidation. In nearly all cases, single amino acid changes were observed to minimally influence the structure and overall properties of β/γ‐crystallins (Guseman et al., 2021; Lampi et al., 2001; Lampi et al., 2006; Pande et al., 2015). Since single Asn to Asp changes appeared to be mostly benign, cumulative influences of multiple changes were speculated to be more important. An increasing number of Asn to Asp and Gln to Glu changes were introduced into γS‐crystallin by the Lampi and Martin groups (Forsythe et al., 2019; Norton‐Baker et al., 2022; Vetter et al., 2020) resulting in varying degrees of stability and folding defects. Most notably, while not affecting the folded protein structure, multiple amino acid changes were shown to alter thermally induced disulfide cross‐linking of γS‐crystallin, increasing the proportion of covalent dimers, which are prone to aggregation (Norton‐Baker et al., 2022). While these findings provided a potential mechanism for γS‐crystallin deamidation‐induced aggregation, the implicated disulfide cluster is only present in γS‐crystallin, being absent in other γ‐crystallins, suggesting that the aberrant intermolecular disulfide‐mediated mechanism for aggregation is γS‐crystallin specific (Norton‐Baker et al., 2022). Therefore, it seemed prudent to investigate the impact of cumulative Asn to Asp changes in other crystallins.

Here, we used structural and biophysical methods to investigate the effect of cumulative Asn to Asp changes in γD‐crystallin. Accounting for nearly 7% of the crystallin protein mass, γD‐crystallin is the third most abundant γ‐crystallin in the human eye lens and is the major component in the lens nucleus (Robinson et al., 2006). Previously, we characterized the effects of single Asn to Asp changes for each of the seven Asn residues in γD‐crystallin and demonstrated that these changes did not alter the overall structure of the protein or its biophysical properties (Guseman et al., 2021). The present study was aimed to assess whether cumulative changes more pronouncedly influence the properties of γD‐crystallin. Therefore, we generated three different protein constructs: (i) all Asn residues in the N‐terminal domain were changed to Asp, termed N24D‐N33D‐N49D‐γD‐crystallin or N‐less NTD, (ii) all Asn residues in the C‐terminal domain were changed to Asp, termed N118D‐N124D‐N137D‐N160D or N‐less CTD, and (iii) fully Asn to Asp γD‐crystallin protein, termed N‐less γD‐crystallin, and we structurally and biophysically characterized all three protein variants.

## 2 RESULTS

**FIGURE 1:**
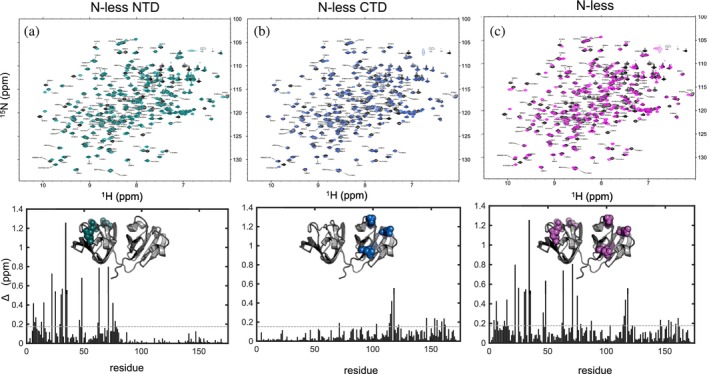
Top panels: Superpositions of ^1^H‐^15^N HSQC spectra of γD‐crystallin N‐less NTD (a, teal), N‐less CTD (b, blue), and N‐less (c, magenta) γD‐crystallin variants and WT γD‐crystallin (a, b, c, black). Bottom panels: Chemical shift differences between γD‐crystallin N‐less NTD, N‐less CTD, and N‐less γD‐crystallin variants and WT γD‐crystallin; the dashed lines are drawn at one standard deviation of Δ*δ*. The respective Asp residues in the deamidation variants are shown in space filling representation on backbone ribbon models of γD‐crystallin. WT, wild‐type (PDB ID 1HK0).

In order to evaluate whether multiple Asn to Asp replacements change the structure of γD‐crystallin, ^1^H‐^15^N HSQC “fingerprint” spectra were recorded for each domain variant and the fully N‐less protein (Figure 1). All spectra exhibit well dispersed resonances of uniform intensity, and no minor resonances are present. This indicates that all variants are stably folded into a single structure, without the presence of minor conformers. The overall appearance of the spectra is comparable to that of wild‐type (WT) γD‐crystallin, suggesting that only minimal structural changes are induced by the amino acid changes. This permitted easy and unambiguous transfer of resonance assignments. Chemical shift perturbation (CSP) analysis between N‐less NTD, N‐less CTD, and WT γD‐crystallin (Figure 1) revealed that all notable chemical shift changes are associated with the positions of the amino acid changes and the local regions around them, primarily confined to the domains that harbored the changes. Furthermore, they are very similar to CSPs observed in previously characterized single Asn to Asp variants. The size of the chemical shift changes imparted by amino acid substitutions in the N‐terminal domain is larger than those in the C‐terminal domain, similar to previous observations with single amino acid changes. Importantly, changes in the N‐ or C‐terminal domains do not propagate across domain boundaries. Accordingly, the fully N‐less γD‐crystallin exhibits CSPs throughout both domains and the CSPs correspond to the sum of the CSPs observed for each individual domain (Figure S1 in Data S1).

**FIGURE 2:**
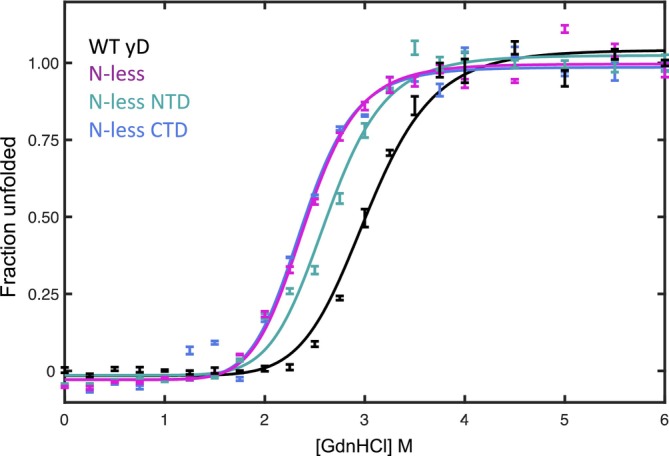
Guanidinium HCl unfolding isotherms of γD‐crystallin variants.

To further investigate whether Asn to Asp changes result in any significant thermodynamic stability changes in the γD‐crystallin variants, compared to WT γD‐crystallin, we measured protein unfolding by guanidinium hydrochloride (GdnHCl), monitored by tryptophan fluorescence (Figure 2). γD‐crystallin has four tryptophan residues, two in each domain that are buried in the hydrophobic core and thus are sensitive to unfolding of the protein. Unfolding started at ~2 M GdnHCl, and at ~4 M GdnHCl, complete unfolding was reached. Fitting all data to a two‐state model, a transition midpoint concentration of 3 ± 0.1 M GdnHCl, an *m*‐value (the dependence of Δ*G*°′_U_ on denaturant concentration) of 2400 ± 300 kcal/mol/M, and a free energy of unfolding (Δ*G*°′_U_) of 7.4 ± 0.8 kcal/mol was extracted for WT γD‐crystallin, consistent with previously reported parameters (Ji et al., 2013). For all deamidation variants, unfolding curves could be fit by a two‐state model, and thermodynamic parameters are listed in Table 1. The N‐less CTD and the fully N‐less γD‐crystallin were equally destabilized, exhibiting transition midpoint concentrations of 2.4 M GdnHCl and Δ*G*°′_U_ values of 4.6 ± 0.9 kcal/mol. The N‐less NTD variant was also destabilized, albeit slightly less, with a transition midpoint concentrations of 2.6 M GdnHCl and a Δ*G*°′_U_ of 4.9 ± 0.9 kcal/mol. While all Asn to Asp γD‐crystallin variants were distinctly different in stability from WT γD‐crystallin, the Δ*G*°′_U_ values for all three lie within the uncertainty of each other (Table 1).

**TABLE 1:** Thermodynamic parameters extracted from GdnHCl unfolding isotherms (transition midpoint, Δ*G*°′_U_, *m*‐value) and DIP of γD‐crystallin and the N‐less variants.

Protein	Midpoint (M GdnHCl)	Δ*G*°′ (kcal/mol)	*m*‐value (kcal/mol/M)	DIP (mL/g)
Wild‐type	3.0 ± 0.1	7.4 ± 0.8	2400 ± 300	−3 ± 1
N‐less	2.4 ± 0.1	4.6 ± 0.9	1900 ± 400	−4 ± 1
N‐less NTD	2.6 ± 0.1	4.9 ± 0.9	1800 ± 400	−5 ± 1
N‐less CTD	2.4 ± 0.1	4.6 ± 0.9	1900 ± 500	−5 ± 1

Abbreviations: DIP, diffusion interaction parameter; GdnHCl, guanidinium hydrochloride.

**FIGURE 3:**
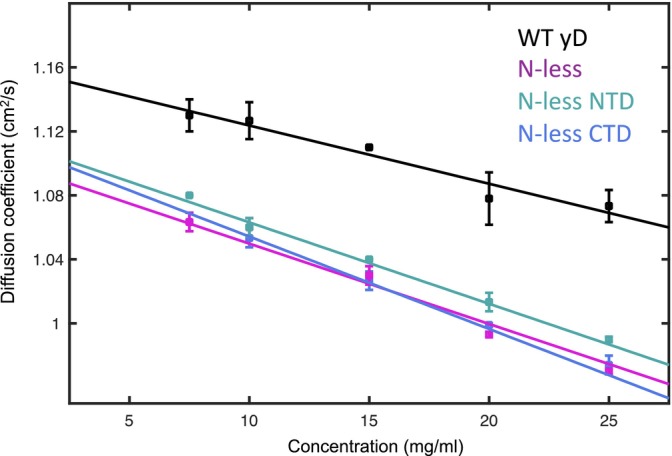
Diffusion interaction parameters of WT γD‐crystallin and all three Asn to Asp variants.

To investigate colloidal stability of the N‐less deamidation variants, dynamic light scattering (DLS) was used to measure the diffusion interaction parameter (DIP). Similar to the second viral coefficient, positive DIPs report on weak repulsive interactions and negative DIPs on weak attractive interactions (Saluja et al., 2010). For WT γD‐crystallin, we obtained a DIP of −3 ± 1 mL/g, consistent with our previously reported data (Guseman et al., 2021) (Figure 3). The N‐less NTD and N‐less CTD both yielded DIP values of −5 ± 1 mL/g, while the fully N‐less variant yielded a DIP of −4 ± 1 mL/g. Thus, DIP analysis reveals that the weak attractive interactions between γD‐crystallins are not significantly different between the Asn to Asp variants, although a clear difference from WT γD‐crystallin is noted.

**FIGURE 4:**
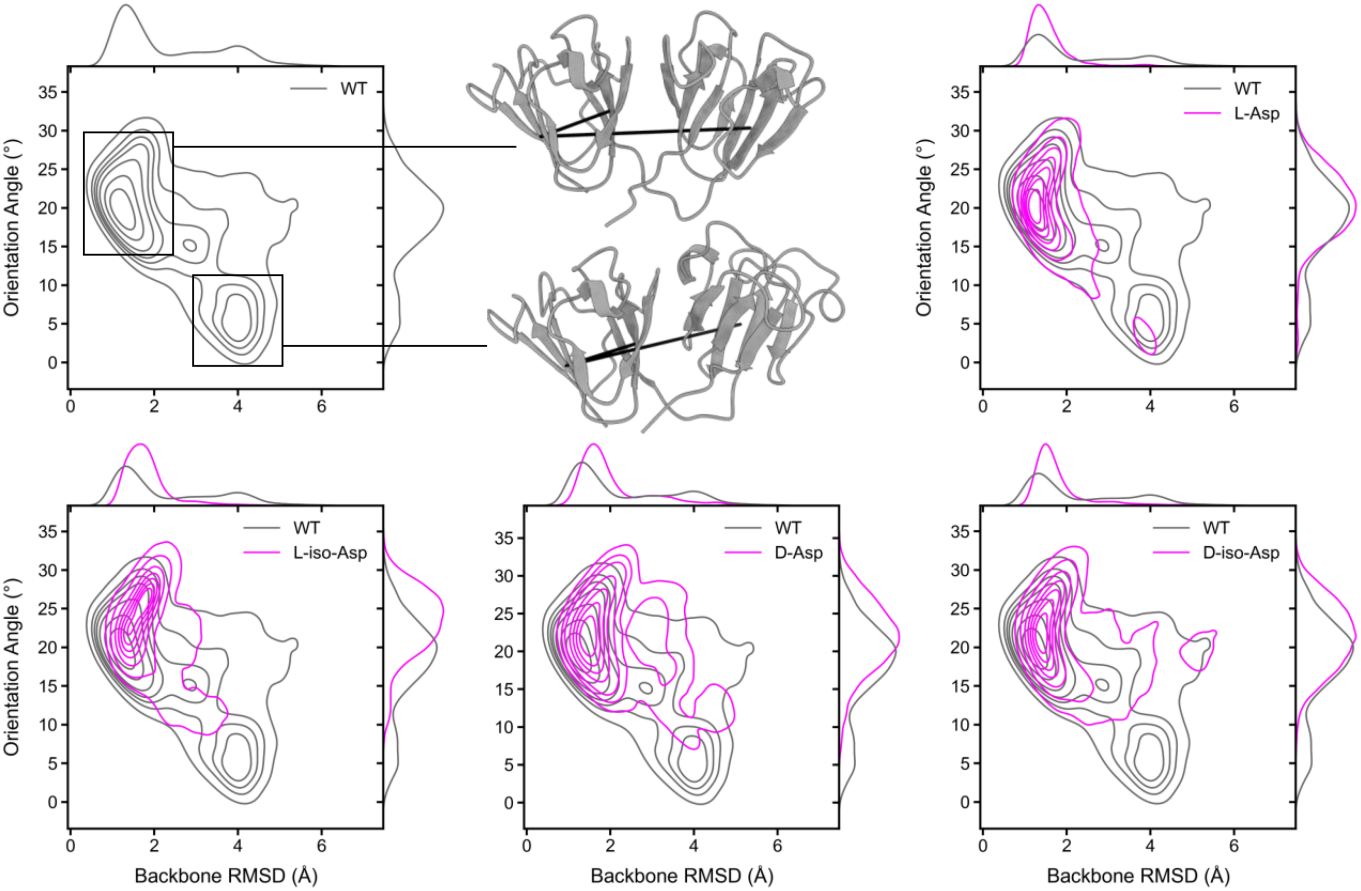
Probability distributions comparing molecular dynamics simulations of WT γD‐crystallin (gray contours) and the all L‐Asp, all D‐Asp, all L‐iso‐Asp, and all D‐iso‐Asp γD‐crystallin variants (magenta contours). Kernel density estimations are projected onto the margins of each joint plot. Representative snapshots of the WT structures (in ribbon representation) of the major and minor conformational states are shown in the upper middle panel, and these are associated with conformational wells (boxed) in the upper left and lower right of the WT contour map, respectively. The vectors that define the orientation angle are shown as black lines within the conformation snapshots.

To explore whether Asn to Asp changes in γD‐crystallin affect short timescale dynamics and conformational ensembles, we carried out 125 μs of aggregate molecular dynamics (MD) simulations for WT γD‐crystallin and the all L‐Asp, D‐Asp, L‐iso‐Asp, or D‐iso‐Asp γD‐crystallin N‐less variants. We projected the conformational landscape of each variant onto a probability distribution of the backbone root‐mean‐square deviation (RMSD) in reference to the crystal structure of WT γD‐crystallin (PDB ID: 1HK0) and an angle that tracks the orientation of the CTD and NTD relative to each other (orientation angle) (Figure 4). The contour plots represent the frequency or probability of all the protein conformations we observed in our simulations. The contour peaks indicate densely sampled regions, correlating to stable conformational states in the γD‐crystallin ensemble.

Our simulation results demonstrate that all N‐less variants sample major conformational ensembles similar to WT γD‐crystallin, within <2 Å in backbone RMSD and around a 20° orientation angle. WT γD‐crystallin also populates a minor conformation at around 4 Å backbone RMSD and <10° orientation angle, as well as a metastable transition state between these two conformations at around 2.8 Å backbone RMSD and 15° orientation angle. In the minor, alternate state, the overall fold of each domain remains intact, while the orientation between the two domains is changed, likely propagated by dynamics within the flexible linker between the domains (Video S1 in Data S1). Although the minor conformation is less frequently sampled by the all L‐Asp γD‐crystallin variant (Video S2 in Data S1), the actual interconversion pathway is the same as observed in WT γD‐crystallin (Figures S2 and S3 in Data S1). For all D‐Asp, all L‐iso‐Asp, and all D‐iso‐Asp γD‐crystallin variants, we observe sampling of some of the same >2 Å RMSD conformational space as seen in WT γD‐crystallin. While the D‐Asp and both iso‐Asp γD‐crystallin variants can sample the metastable intermediate, they cannot sample the minor conformation to the same extent as the WT γD‐crystallin (Figures S4–S6 in Data S1).

We examined the domain rearrangement in the minor conformation in greater detail by tracking the distances between the Asn to Asp substitution sites as well as the distances between each substitution site and each negatively charged residue within N‐less γD‐crystallin (Figure S7 in Data S1). To do so, we used a single N‐less trajectory that transitioned from the major to the minor conformation and compared the two points in the trajectory that correspond to each conformation. When only considering the Asn to Asp substitution distances (Figure S7A in Data S1), no strong differences in intra‐domain distances are observed (Figure S7B in Data S1), while for inter‐domain distances, the D49 to D124 distance is different between the two conformations at a 20 Å distance cutoff. Because this interaction is around 11 Å apart, it most likely is not responsible for the change in population of the minor conformation. To better characterize all potential interactions involving individual Asn to Asp changes, we also compared distances between the newly introduced Asp residues and all negatively charged γD‐crystallin residues in the N‐less variant. Consistently, we see no significant changes in intra‐domain distances. However, within a 15 Å distance cutoff, small distance changes bring D49 closer to D149 and D155 (Figure S7C in Data S1). This repulsive interaction may prevent the transition to the minor population. This contrasts with WT γD‐crystallin where the amino group of N49 has the potential to engage in a favorable contact with D149 and D155 through hydrogen bonding, while in the N‐less γD‐crystallin, the N49D change replaces this stabilizing interaction with a repulsive one (Figure S8 in Data S1).

We previously studied the γD‐crystallin N49D single point variant (Guseman et al., 2021) and found that *T*_m_ and CSPs were almost identical to the WT data. This indicates that the N49D variant, which is likely able to modulate the propensity of the minor conformation, does not have any effect on overall protein stability and fold. Since the domain rearrangements seen in our simulation data occur faster than within 1 μs, they will not influence the chemical shifts.

Overall, our simulation data show that all of the γD‐crystallin variants sample the same major stable conformation as WT, exploring a similar, but slightly more restricted conformational space. Our data also suggest that the introduction of charge–charge repulsions caused by introducing three and four negative charges in the NTD and CTD, respectively, may restrict the transition to the minor conformation, exemplified by the N49D change that introduces a repulsive force.

## 3 DISCUSSION

Structural, dynamics, and biophysical properties of crystallin proteins hold important keys for understanding their proteostasis in the eye lens. In the present study, we investigated whether cumulative Asn to Asp changes, mimicking one of the outcomes of deamidation, induce structural and/or other changes in the biophysical properties of human γD‐crystallin. To that end, we replaced every Asn residue with Asp in γD‐crystallin, generating a fully N‐less γD‐crystallin variant, as well as N‐Less NTD and N‐Less CTD variants.

Using ^1^H‐^15^N HSQC spectra as a structural fingerprint, we demonstrate that Asn to Asp deamidation, in either the N‐terminal domain, the C‐terminal domain, or the entire protein minimally perturbs the overall protein structure, given that all spectra are very similar. Upon mapping all CSPs for these deamidation variants, we note that they are locally limited to the position of the change in the sequence and comparable in magnitude to the individual Asn to Asp variants that we previously investigated (Guseman et al., 2021). For example, CSPs are noted in the N‐less NTD variant near position 33 as well as around E7 and D73. These shift differences were also observed for the single N33D γD‐crystallin variant, and can be attributed to the disruption of a hydrogen bonding network. In particular, replacement of the Asn that acts as a hydrogen bond donor to the E7 and D73 carboxylate side chains seems to play a role (Guseman et al., 2021). Furthermore, CSPs observed in the fully N‐less γD‐crystallin are nearly identical to the sum of the observed perturbations for the N‐less NTD plus the N‐less CTD (Figure S1 in Data S1). Therefore, our NMR‐derived structural assessment unequivocally demonstrates that cumulative Asn to Asp deamidation does not significantly alter the structure of human γD‐crystallin.

We also evaluated whether Asn to Asp deamidation of human γD‐crystallin results in any differences in thermodynamic or colloidal stability, properties that are frequently evoked as triggering cataract formation. GdnHCl unfolding experiments revealed a 2.5–2.8 kcal/mol, destabilization for the N‐less NTD, N‐less CTD, and N‐less γD‐crystallin variants, compared to WT γD‐crystallin (Figure 2). Such small destabilization is not unexpected for variants in which three, four, or seven amino acids are replaced, especially for changes that alter the overall charge of the protein. Deamidation of γD‐crystallin is detected by proteomics on five of seven Asn residues, however in aged lenses three Asn residues (N49, N137, and N160) are predominantly deamidated (Hains and Truscott, 2010). Given that the all Asn to Asp deamidation variant represents an extreme case of deamidation and that this N‐less variants' stability is not dramatically affected, we need to call into question the often‐advanced notion that deamidation severely destabilizes the folded structure and is a cause for cataract. In addition, our DIP analysis demonstrates that all N‐less variants yielded DIP values very similar to WT γD‐crystallin. Therefore, Asn to Asp deamidation is not altering the colloidal stability and, vide infra, not their aggregation propensities (Figure 3).

In the native environment of the eye lens, deamidation occurs slowly and requires the Asn sidechain to undergo a condensation reaction, in which a cyclic succinimide intermediate is formed with the N + 1 amide (Robinson, 2002). Subsequent hydrolysis of the succinimide into Asp or iso‐Asp occurs, generating both the L‐ and D‐isomers of the Asp or Iso‐Asp products. In most in vitro studies of deamidation to date, L‐Asn to L‐Asp changes are used as mimics of deamidation, given the ease with which these protein variants can be generated by molecular biology. Most studies show that the L‐Asp product of deamidation is minimally perturbing to protein structure and biophysical properties. In order to site‐specifically generate iso‐Aspartate changes in proteins, chemical synthesis approaches are necessary, which are still challenging for sizable proteins, and further development is needed. Therefore, very little data on the influence of iso‐Asp on protein structure is available. We therefore addressed computationally whether Asn to Asp and iso‐Asp changes alter the dynamical behavior of γD‐crystallin by performing MD simulations that report on μs timescale motions, using appropriate force fields in which modifications for nonnatural amino acids such as iso‐aspartate have been introduced (Figure 4). Here again, our results indicate that both the WT γD‐crystallin and the N‐less γD‐crystallin variants sample similar major conformations, and, compared to WT γD‐crystallin, only minimal influences on the conformational landscapes were observed for all Asn to L‐Asp, D‐Asp, L‐iso‐Asp, or D‐iso‐Asp γD‐crystallin variants.

Thus, overall, our combined experimental and computational data on Asn to Asp variants demonstrate that cumulative Asn to Asp deamidation minimally alters the structure, dynamics, or other biophysical properties of γD‐crystallin. Our results also highlight that each γ‐crystallin exhibits distinct individual properties, despite possessing similar 3D structures and being subjected to similar posttranslational modifications and/or chemical damage (Forsythe et al., 2019; Norton‐Baker et al., 2022; Pande et al., 2015; Ray et al., 2016; Takemoto and Boyle, 2000; Vetter et al., 2020). For example, cumulative Asn to Asp and Gln to Glu changes in γS‐crystallin rendered this crystallin susceptible to oxidative stress (Vetter et al., 2020) and disulfide‐mediated aggregation (Norton‐Baker et al., 2022), while for γD‐crystallin this does not appear to be the case.

As the lens environment does not undergo protein turnover, deamidation and other PTMs are often considered detrimental to the properties of the native protein. Since PTMs are not encoded in the genome, they are not under evolutionary pressure. In the case of crystallins, and in particular γD‐crystallin that was studied here, only minimal effects on structure and thermodynamics are seen. This finding, combined with the fact that many crystallin modifications appear in age‐matched lenses from both cataractous lenses and normal lenses, prompts reconsideration of whether these changes are truly “damaging” or simply bystander changes that occur in long‐lived proteins (Hains and Truscott, 2010). PTMs could be benign until a critical number of PTMs accumulate, and it is the additive effect of many minor changes that, in their totality, are detrimental (Carver et al., 2018; Grosas and Carver, 2021; Grosas et al., 2021; Quinlan and Clark, 2022; Schmid et al., 2021).

## 4 CONCLUSIONS

Deamidation, as exemplified by Asn to Asp changes, is a common modification found in crystallins by proteomic analysis of eye lenses, especially aged lenses, and is often postulated as a cause of cataract formation. Given the above results, where does that leave our understanding of the relationship between asparagine deamidation and cataract? For human γD‐crystallin, based on our extensive studies of Asn to Asp deamidation over the last decade, only minimal influences on the structure, thermodynamic stability, colloidal stability, and dynamics of such variants have been observed, whether the changes involved individual single amino acid positions or were cumulative in nature. Even for the extreme cases of all D‐ or L‐iso‐Asp variants, which could only be interrogated computationally, the current MD study did not provide any compelling evidence for substantive differences. We therefore suggest that, at least for human γD‐crystallin, Asn to Asp deamidation is likely not the major driver of cataract formation, suggesting that additional posttranslational damage likely is at play.

## 5 MATERIALS AND METHODS

### 5.1 Protein expression and purification

γD‐crystallin and its variants were expressed and purified as previously described (Guseman et al., 2021). In short, single colonies of *E. coli* BL21DE3 cells harboring plasmids encoding WT γD‐crystallin (pET14b‐derived) or its Asn to Asp mutants (pET28b‐derived) were used to inoculate 50 mL of Luria Bertani broth and cultures were grown over night at 37°C; 10 mL of each overnight culture was used to inoculate 1 L of modified M9 minimal medium with ^15^NH_4_Cl as the sole nitrogen source. Cells were grown at 37°C with shaking at 225 rpm to an OD_600_ of 0.6, at which point protein production was induced with 1 mM IPTG for 4 h. Cells were harvested by centrifugation at 3500 × *g* for 20 min, the cell pellet was resuspended in Q_a_ buffer (20 mM Tris, pH 8.0, 1 mM EDTA, 1 mM TCEP), and cells were lysed using a microfluidizer. The lysate was centrifuged at 18,000 × *g* for 45 min, the supernatant was passed through a 0.22 mm filter and loaded onto Q HP anion exchange column. For the WT, N‐less NTD and N‐less CTD γD‐crystallin variants, the flowthrough from the HP Q column was collected, dialyzed overnight in 3 L of S_a_ buffer (20 mM MES at pH 6.0, 1 mM EDTA, 1 mM TCEP), and centrifuged at 18,000 × *g* for 30 min to remove any precipitate. The supernatant was loaded onto a HP SP cation exchange column, preequilibrated in S_a_ buffer, and protein was eluted using a gradient of 0%–100% S_b_ (20 mM MES 1 mM EDTA, 1 mM TCEP,1 M NaCl, pH 6.0). Protein‐containing fractions were pooled and concentrated to 1 mL, loaded onto a S75 16/60 column, preequilibrated with S_a_ buffer, and further fractionated over one column volume of S_a_. Crystallin‐containing fractions were pooled, concentrated to <1 mL, and purity was ascertained by SDS‐PAGE and mass spectrometry. For the fully N‐less γD‐crystallin, the protein was loaded onto a Q HP column and eluted with a gradient of 0%–100% Q_B_ (20 mM Tris, pH 8.0, 1 mM EDTA, 1 mM TCEP, 1 M NaCl, pH 7.5). Protein fractions were collected, pooled, and concentrated to 1 mL, loaded onto a S75 16/60 column, preequilibrated with S_a_ buffer, and eluted over one column volume of S_a_. γD‐crystallin‐containing fractions were collected, pooled, and concentrated to <1 mL, analyzed by SDS‐PAGE and mass spectrometry to confirm the identity and purity of the protein.

### 5.2 NMR spectroscopy

All γD‐crystallin samples contained 300 μM protein in 20 mM MES, 1 mM EDTA, 1 mM TCEP pH 6.0 buffer, and 10% D_2_O. Spectra were recorded at 25°C on a Bruker AVANCE III 600 MHz spectrometer, equipped with a *z*‐axis gradient triple resonance cryoprobe. 2D ^1^H‐^15^N HSQC spectra were recorded with 16 scans and 256 increments. Spectra were analyzed and resonances were assigned using POKY (Lee et al., 2021). CSPs were calculated according to Equation (1) (Williamson, 2013) and plotted in MATLAB.

(1) $$∆\delta =\sqrt{{\left(\delta H_{V}-\delta H_{\mathrm{wt}}\right)}^{2}+{\left(\frac{\delta N_{v}-\delta N_{\mathrm{wt}}}{6}\right)}^{2}\;}.$$

### 5.3 DLS

Eighty microliters of purified γD‐crystallin samples were prepared with concentrations ranging from 5 to 25 mg/mL in 20 mM MES, pH 6.0, 1 mM EDTA, and 1 mM TCEP. Samples were passed through 0.01 μM syringe filters, and concentrations were confirmed by measuring UV absorbance at 280 nm with a molar extinction coefficient of 40,680; 20 μL of each sample were loaded in triplicate on a 384‐well plate, and 10 acquisitions were measured for each well. Data were collected in triplicate, analyzed in dynamics 7.1.7, and fit to *D* = (*D*_0_ + *D*_0_ *k*_d_ *C*) in MATLAB (Saluja et al., 2010). Uncertainties are reported as the 95% confidence interval of the fit.

### 5.4 Unfolding experiments

Individual samples of 10 μM WT γD‐crystallin or N‐less variants in 0–6 M GdnHCl were prepared in 20 mM MES, 1 mM EDTA, 1 mM TCEP, pH 6.0. Samples were equilibrated overnight at 25°C, and 20 μL of each sample were loaded onto a 384‐well plate. Tryptophan fluorescence was recorded on a Tecan Spark, using 295 nM excitation and 320 and 360 nM emission wavelengths. Measurements were carried out in triplicate and averaged for each variant. Data were subsequently processed in MATLAB, normalized and fit to a two‐state unfolding model, extracting *C*‐values, *M*‐values, and Δ*G*°′_U_ (Street et al., 2008). Uncertainties are reported as the 95% confidence interval of the fit.

### 5.5 MD simulations

Atomic coordinates of WT γD‐crystallin (PDB: 1HK0 (Basak et al., 2003)) were used to generate those of the Asn‐less variants replacing Asn to Asp at positions 24, 33, 49, 118, 124, 137, and 160 using the rotamers tool (Shapovalov and Dunbrack, 2011) in ChimeraX (Pettersen et al., 2004). All Asp stereocenters were switched from L to D to build the all D‐Asp variant. For the all L‐iso‐Asp variant, each residue was built manually using ChimeraX, with the updated atom names adjusted to match those in the AMBER ff15ipq‐m force field for unnatural amino acids (Bogetti et al., 2020). For the all D‐iso‐Asp variant, the L‐iso‐Asp stereocenters were switched from L to D. Since the ff15ipq force field does not contain correlated F and Y dihedral correctional profiles (CMAP), the Asp stereo inversion should be compatible with the same force field parameters (Oda et al., 2018; Zou et al., 2016). Side chain protonation states for all ionizable residues were adjusted to represent the major species present at the experimental pH of 6.0. Simulations were performed using the GPU implementation of the PMEMD module of the AMBER 20 software package (Case et al., 2005; Salomon‐Ferrer et al., 2013) and the AMBER ff15ipq and ff15ipq‐m force fields (Bogetti et al., 2020; Debiec et al., 2016). Each system was solvated in a truncated octahedral box of explicit SPC/E_b_ (Takemura and Kitao, 2012) water molecules, with a minimum solute–wall distance of 12 Å. All unpaired charges were neutralized with Na^+^ or Cl^−^ ions and treated with Joung and Cheatham ion parameters (Joung and Cheatham III, 2008). Each system was initially subjected to energy minimization, followed by a three‐stage equilibration. The first two stages involved solvent equilibration in the presence of solute heavy‐atom positional restraints, using a harmonic potential with a force constant of 1 kcal/(mol Å^2^). In the first stage, a 20 ps simulation was carried out at constant volume and temperature. In the second stage, a 1 ns simulation was carried out at constant temperature and pressure. For the third stage, an unrestrained 1 ns simulation was carried out at constant temperature and pressure. Finally, a 1 μs production simulation was carried out at constant temperature and pressure. Overall, the entire equilibration and simulation process was repeated 25 times for the WT γD‐crystallin, all L‐Asp, all D‐Asp, all L‐iso‐Asp, and all D‐iso‐Asp variants, yielding over 125 μs of aggregate production simulation time. To enable a 2 fs time step, all CH and NH bonds were constrained to their equilibrium values using the SHAKE algorithm (Ryckaert et al., 1977). Simulation temperatures were maintained at 298 K using a Langevin thermostat with a frictional constant of 1 ps^−1^, while pressure was maintained at 1 atm using a Monte Carlo barostat with 100 fs between system volume changes. Van der Waals and short‐range electrostatic interactions were truncated at 10 Å, while long‐range electrostatic interactions were calculated using the particle mesh Ewald method (Essmann et al., 1995). Coordinates were saved every ps, and analysis was performed in CPPTRAJ (Roe and Cheatham III, 2013). The RMSD of the backbone heavy atoms was calculated with reference to the crystal structure of WT human γD‐crystallin (PDB: 1HK0). The orientation angle was measured between two vectors defined by the center‐of‐mass of the backbone heavy atoms of residues 43 and 131 with a vertex at the center‐of‐mass of residues 4 and 36. The distance matrices were calculated based on the center‐of‐mass distance between residues, considering both backbone and sidechain atoms. Representative PDB snapshots, along with simulation setup and analysis files, are openly available through GitHub: https://github.com/darianyang/crystallin.

## AUTHOR CONTRIBUTIONS

**Alex J. Guseman:** Conceptualization; investigation; methodology; writing – original draft; writing – review and editing; supervision; data curation; project administration; formal analysis; visualization. **Jeremy J. González:** Investigation; writing – review and editing; data curation; methodology. **Darian Yang:** Investigation; writing – review and editing; formal analysis; data curation; visualization; writing – original draft. **Angela M. Gronenborn:** Conceptualization; funding acquisition; writing – review and editing; project administration; supervision; data curation; formal analysis; writing – original draft.

## Supporting information


**Data S1.** Supporting information.
