# Supplementary material for: Cumulative asparagine to aspartate deamidation fails to perturb γD‐crystallin structure and stability
Source: Protein Sci. 2024 Jul 18;33(8):e5120. doi: 10.1002/pro.5120 (PMC11255865; doi:10.1002/pro.5120)
Supplement: Supplementary file 1 — Data S1. Supporting information. [file PRO-33-e5120-s001.docx]

Supporting information for

Cumulative Asparagine to Aspartate Deamidation Fails to Perturb γD-Crystallin Structure and Stability

Alex J Guseman^1^*, Jeremy J. González^1^*, Darian Yang^1^, Angela M. Gronenborn^1,2^

Figure S1:

Figure S1: Chemical shift differences between γD-crystallin N-less NTD, N-less CTD, and N-less γD-crystallin variants and WT γD-crystallin (top). The respective Asp residues in the deamidation variants are shown in space filling representation in backbone ribbon models of γD-crystallin. In the bottom panels differences between the overall chemical shift differences and the domain-specific chemical shift differences are shown; C-A in D; C-B in E and C- (A+B) in F.


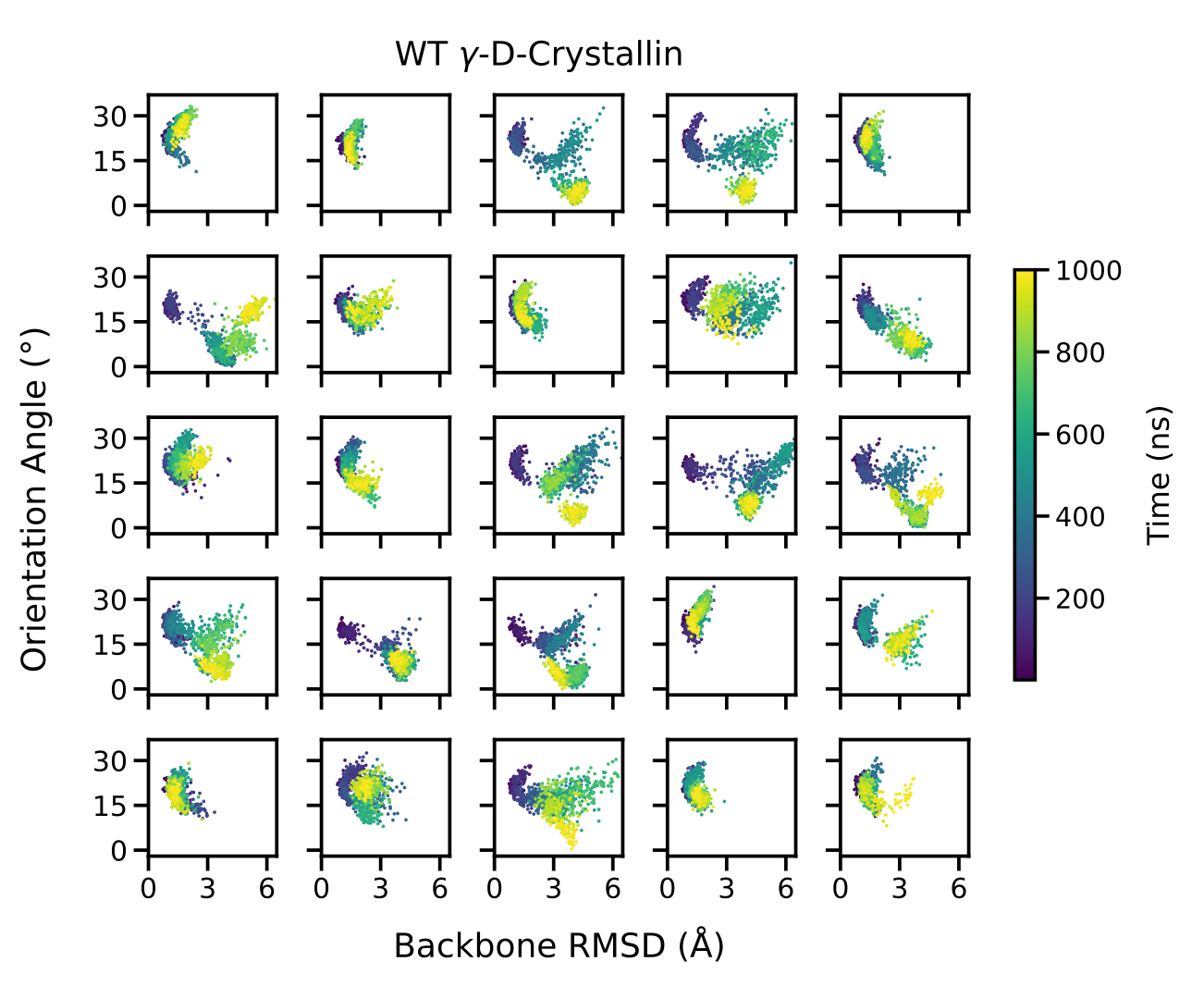


Figure S2: Representative 1 µs pathways in the MD conformational landscape of WT γD-crystallin for 25 replicates. The color of each point in the scatter plot conveys time course information on the time scale on the right side.

Figure S3: Representative 1 µs pathways in the MD conformational landscape of the all L-Asp γD-crystallin for 25 replicates. The color of each point in the scatter plot conveys time course information on the time scale on the right side.

Figure S4: Representative 1 µs pathways in the MD conformational landscape of the all D-Asp (N-less) γD-crystallin for 25 replicates. The color of each point in the scatter plot conveys time course information on the time scale on the right side.


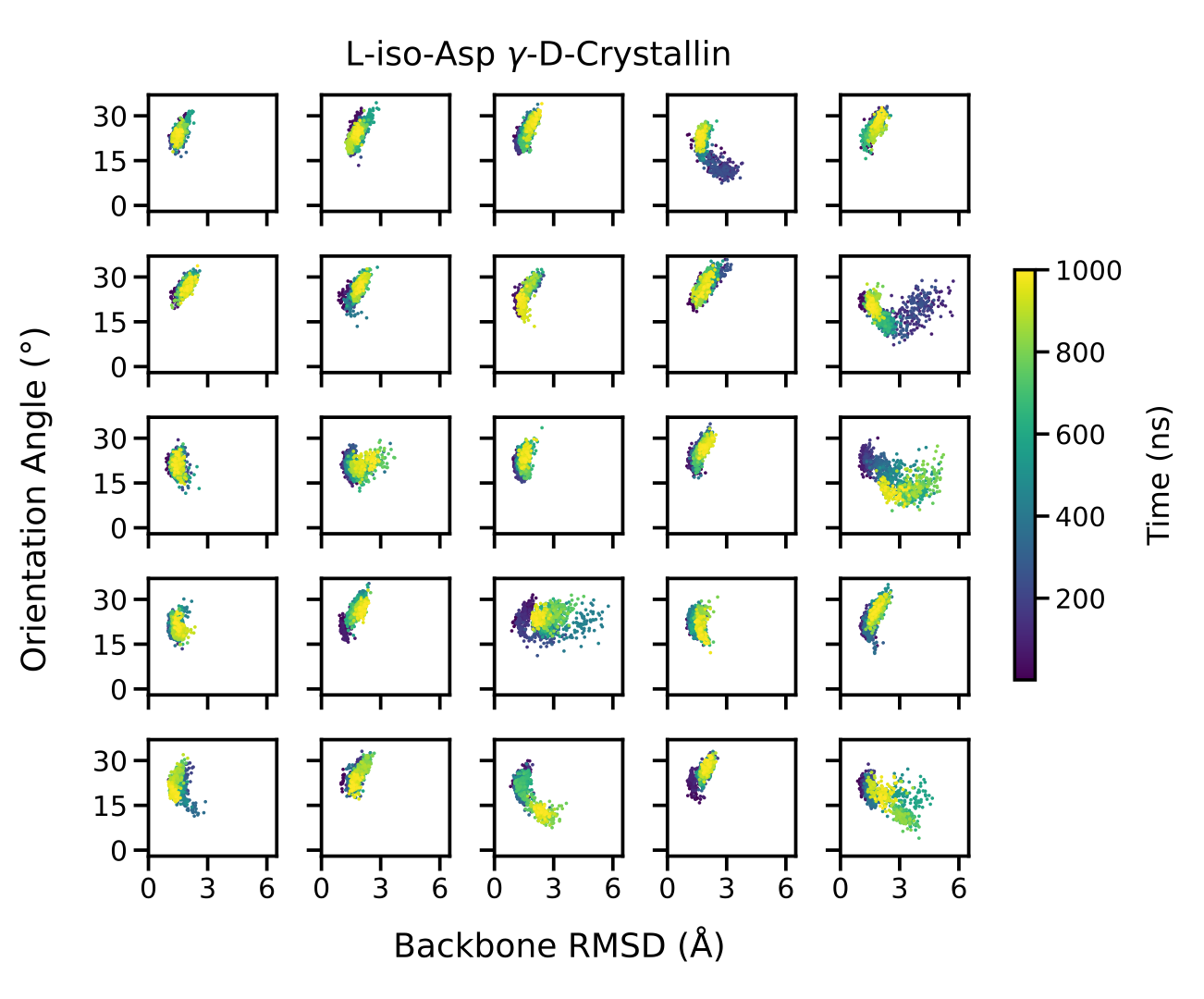


Figure S5: Representative 1 µs conformational pathways in the MD conformational landscape of all L-iso-Asp γD-crystallin for 25 replicates. The color of each point in the scatter plot conveys time course information on the time scale on the right side.


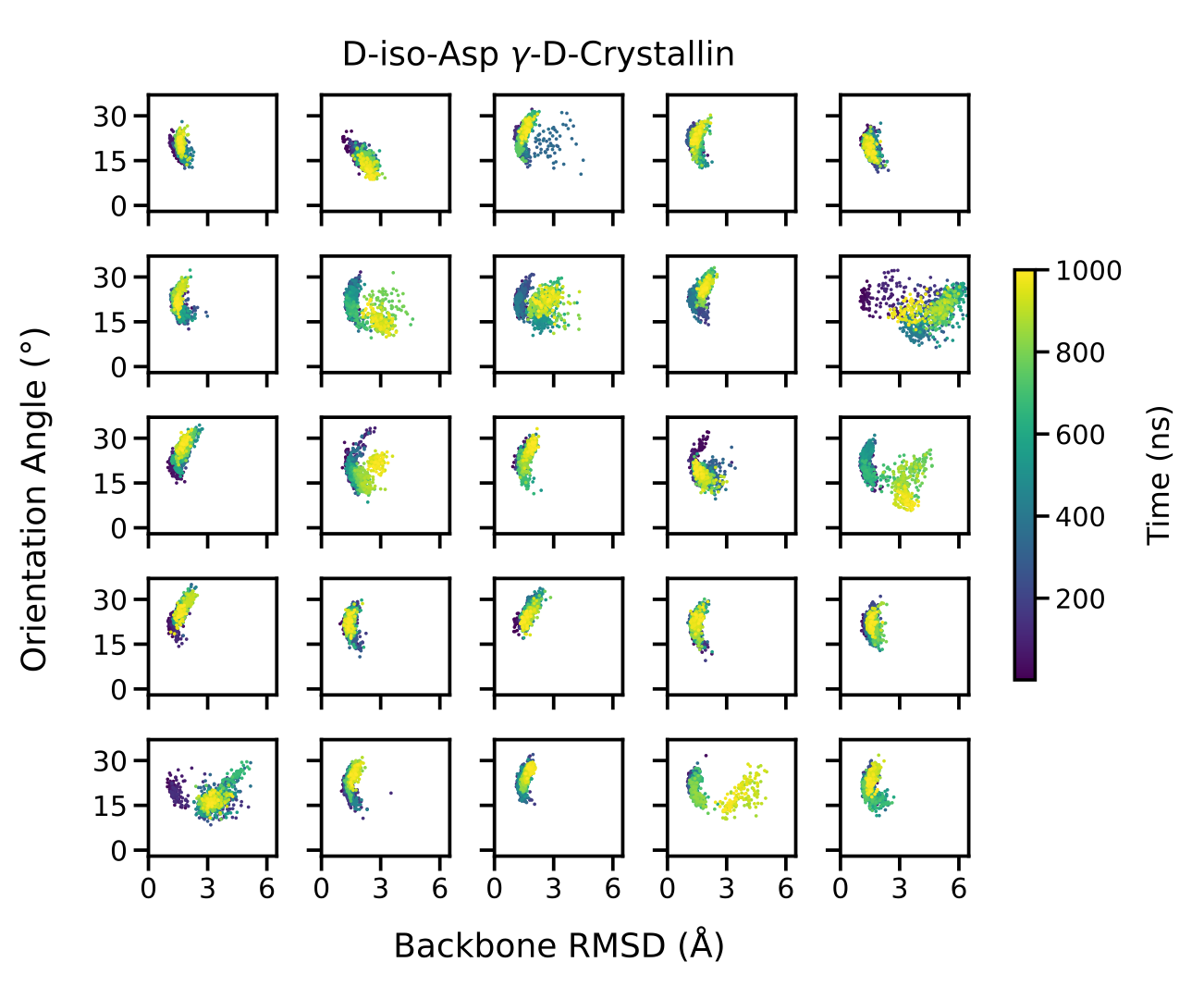


Figure S6: Representative 1 µs pathways in the MD conformational landscape of the all D-iso-Asp γD-crystallin for 25 replicates. The color of each point in the scatter plot conveys time course information on the time scale on the right side.

Figure S7: Distance matrix plots of the γD-crystallin Asn to Asp changes relative to each other (A, B) and of the γD-crystallin Asn to Asp replacements relative to all Asp and Glu residues (C). Distances were derived from a representative N-less γD-crystallin MD trajectory which simulated conformational changes from the major (100ns) to minor (1000ns) conformation. Each distance matrix plot represents intra- and inter-domain distances. The difference plots (B, bottom panel of C) show that the intra-domain distances remain constant while there are a few key differences for inter-domain distances. Note, distance cutoffs are used since we are focusing on identifying potential close-range electrostatic interactions. Distance outliers are in black boxes to highlight key inter-domain residues whose distance changed from the major to minor γD-crystallin conformation.

Figure S8: Representative structures of the major and minor conformations for WT and N-less γD-crystallin. Spherical atomic representations and labeling are added for the key residues that change in distance between the major to minor conformation, as identified from the distance matrix plots (Figure S6).


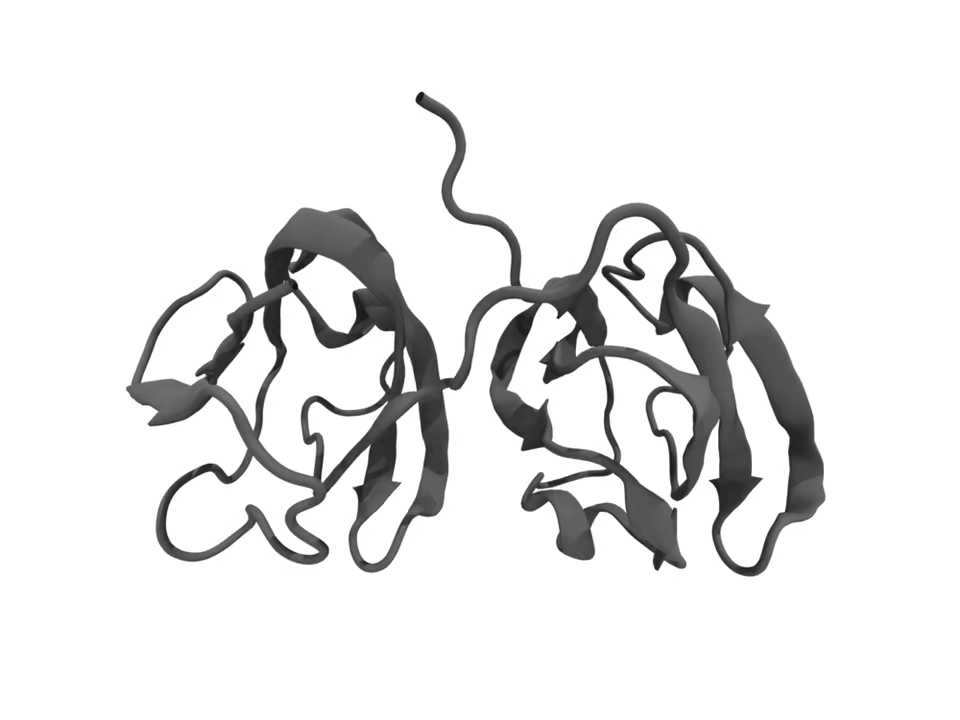


Video S1: A representative 1 µs MD trajectory of the interconversion of WT γD-crystallin from the major to minor conformation.


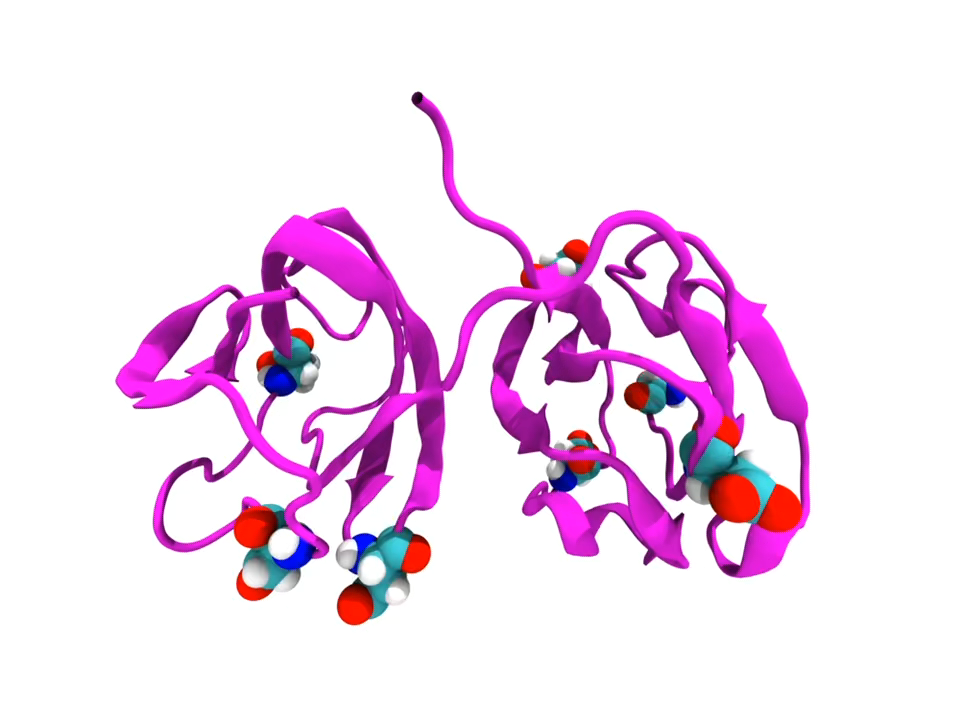


Video S2: A representative 1 µs MD trajectory of the interconversion of N-less γD-crystallin from the major to minor conformation. Each Asn to Asp position is shown in van der Waals sphere representation.
